# Small molecule analogues of the immunomodulatory parasitic helminth product ES-62 have anti-allergy properties

**DOI:** 10.1016/j.ijpara.2014.05.001

**Published:** 2014-08

**Authors:** Justyna Rzepecka, Michelle L. Coates, Moninder Saggar, Lamyaa Al-Riyami, Jennifer Coltherd, Hwee Kee Tay, Judith K. Huggan, Lucia Janicova, Abedawn I. Khalaf, Ivonne Siebeke, Colin J. Suckling, Margaret M. Harnett, William Harnett

**Affiliations:** a Strathclyde Institute of Pharmacy and Biomedical Sciences, University of Strathclyde, Glasgow G4 0RE, Scotland, UK; b Institute of Infection, Immunity and Inflammation, University of Glasgow, Glasgow G12 8TA, Scotland, UK; c Department of Pure and Applied Chemistry, University of Strathclyde, Glasgow G1 1XL, Scotland, UK

**Keywords:** Asthma, ES-62, Parasitic helminth, Immunomodulation, Phosphorylcholine

## Abstract

ES-62, a glycoprotein secreted by the filarial nematode *Acanthocheilonema viteae*, exhibits anti-inflammatory properties by virtue of covalently attached phosphorylcholine moieties. Screening of a library of ES-62 phosphorylcholine-based small molecule analogues (SMAs) revealed that two compounds, termed 11a and 12b, mirrored the helminth product both in inhibiting mast cell degranulation and cytokine responses in vitro and in preventing ovalbumin-induced Th2-associated airway inflammation and eosinophil infiltration of the lungs in mice. Furthermore, the two SMAs inhibited neutrophil infiltration of the lungs when administered therapeutically. ES-62-SMAs 11a and 12b thus represent starting points for novel drug development for allergies such as asthma.

## Highlights

• Small molecule analogues of the helminth immunomodulator ES-62 have been produced.
• Two analogues inhibit mast cell functions and prevent airway hypersensitivity.
• The analogues are drug-like and could be considered for treatment of human allergy.

## Graphical abstract

**Figure:**
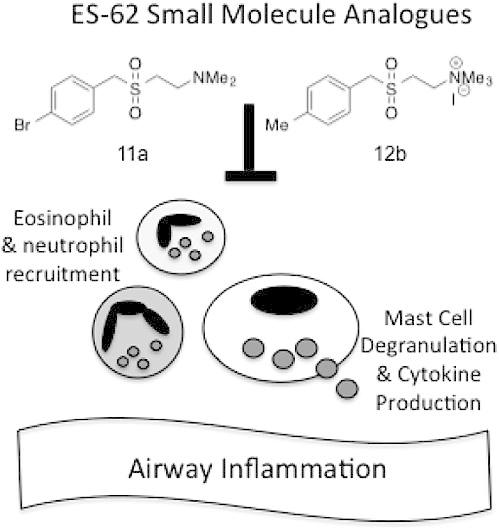

Asthma is a chronic ailment of the lungs associated with airway inflammation and exaggerated responses to infections, allergens and irritants that has shown a clear increase in occurrence in Western countries in recent decades. For example, asthma incidence rose in the USA from 7.3% in 2001 to 8.4% in 2010 such that the number of people afflicted reached 25.7 million, with prevalence highest in children (Akinbami et al., 2012). However, such an increase has not been mirrored in rural areas of the Third World where parasitic worms are highly abundant (see Keiser and Utzinger, 2010) and this has led to the suggestion that such pathogens may protect humans from developing asthma. Although the situation is not clear-cut and may be influenced by factors such as the species of helminth under study, there is sufficient supportive evidence for this idea to possess some merit (reviewed by Cooper, 2009). Furthermore, an increasing body of data exists to show that parasitic worms or their secreted products can prevent the development of allergic conditions in the lungs in mouse models (reviewed by Danilowicz-Luebert et al., 2011). One such secreted product, ES-62, a phosphorylcholine (PC)-containing glycoprotein derived from the rodent filarial nematode *Acanthocheilonema viteae* (Harnett et al., 1989), can directly interfere with allergic responses by blocking mast cell activation via the high affinity IgE receptor, FcεRI, thereby preventing release of inflammatory mediators (Melendez et al., 2007; Ball et al., 2013). However, as shown in a mouse model of ovalbumin (OVA)-induced airway hyper-reactivity (OAH), it is also able to prevent disease by reversing the Th2 cell polarity that contributes to allergy (Melendez et al., 2007; Rzepecka et al., 2013). These data suggest that ES-62 could exhibit therapeutic potential in human asthma but it is a large and hence potentially immunogenic molecule and thus in theory is likely to be unsuitable for use as a drug. For this reason, we decided to investigate a recently prepared library of drug-like small molecule analogues (SMAs) of ES-62 (Al-Riyami et al., 2013). These focus on the PC moiety of the helminth product, as we have previously shown this to be responsible for many of its anti-inflammatory activities (Harnett et al., 2008).

We first investigated in vitro mast cell responses. FcεRI-mediated mast cell activation (degranulation and cytokine secretion) is dependent on the mobilization of intracellular calcium (Gilfillan and Beaven, 2011; Wu, 2011; MacGlashan, 2012) and consistent with this, we have previously shown that the ES-62-mediated desensitisation of such mast cell function is associated with suppression of calcium signalling in human bone marrow-derived mast cells (BMMCs) and mouse peritoneal-derived mast cells (PDMCs) (Melendez et al., 2007; Ball et al., 2013). We therefore employed Fura-2-acetyoxymethyl ester (Fura-2AM) tracking of calcium mobilization as a screen for testing the ability of ES-62 SMAs (65 in total) to similarly desensitise mouse BMMC responses. Only a small number of the compounds showed any evidence of being able to suppress FcεRI-mediated calcium mobilization, with the sulfones 11a and 12b (Fig. 1A and B), which reduced mobilization to 66.4 ± 3.5% and 62.6 ± 5.5% (mean ± S.E.M.) of the control value, respectively (Fig. 1A and B), being most effective. These two SMAs were therefore selected for further analysis as possible anti-allergy ES-62 mimics; of note, we had previously shown one of them - 11a - to prevent development of collagen-induced arthritis (CIA) in mice (Al-Riyami et al., 2013).

Continuing with mast cells, 11a and 12b were first tested for their ability to inhibit degranulation of these cells when activated via FcεRI. We have recently shown (Ball et al., 2013) that whilst mouse BMMCs produce high levels of eicosanoids, cytokines and chemokines, they make poor degranulation responses to activation via FcεRI. By contrast, PDMCs respond strongly in terms of degranulation, but not cytokine production, following stimulation with antigen, whilst bone marrow-derived connective tissue type mast cells (CTMCs) display an intermediate phenotype. Importantly, ES-62 was able to desensitise FcεRI-signalling in all of these mast cell subsets (Ball et al., 2013). Due to their strong degranulation reaction, PDMCs were employed to test the effects of 11a and 12b on this mast cell response, mediated via FcεRI ligation and crosslinking, and both were found to be able to inhibit it (Fig. 1C and D). Similar results were obtained with the rat basophilic leukaemia cell line RBL-2H3 (results not shown). 11a and 12b were next tested for their ability to mimic ES-62 in inhibiting the TNFα (Fig. 1E and F) and IL-6 (Fig. 1G and H) responses of BMMCs activated via FcεRI (Ball et al., 2013). Both SMAs were found to inhibit secretion of these cytokines and in a dose-dependent manner.

ES-62-mediated inhibition of human BMMC responses is associated with degradation of protein kinase C alpha (PKCα) (Melendez et al., 2007) and subsequent uncoupling of FcεRI from phospholipase D (PLD) and sphingosine kinase (SphK), signals that have been shown to be important in calcium mobilization and degranulation in mast cells (Gilfillan and Beaven, 2011; Spiegel and Milstien, 2011). Similarly, we have shown ES-62-mediated desensitisation of mouse PDMC, BMMC and CTMC degranulation and/or cytokine release to be associated with PKCα degradation (Ball et al., 2013). Thus, to determine whether SMAs 11a and 12b were mimicking this mechanism of action of ES-62, we investigated the effect of these SMAs on PKCα expression using quantitative laser-scanning cytometry (Harnett, 2007). PC conjugated to BSA was used as a positive control (and compared with BSA) for this experiment due to the ability of PC-conjugates such as this to mimic immunomodulation by ES-62 (Harnett et al., 2008; Al-Riyami et al., 2013) and as shown in Fig. 2, both SMAs mirrored PC-BSA in down-regulating expression of PKCα in mouse BMMCs. This was evident not only in the percentage of mast cells positive for PKCα, but also in a reduction of the levels of PKCα expression (mean fluorescence integral; MFI) and in terms of foci of PKCα expression (maximum pixel). The last result suggested that the residual low levels of expression observed in PC-BSA or SMA-treated cells reflect a dispersed distribution throughout the cells, rather than translocation and association in foci with membranes, which would be indicative of activation.

SMAs 11a and 12b were next tested for their ability to mimic ES-62 in an in vivo situation, specifically in demonstrating protection against allergic responses in the lungs of mice sensitised and challenged with OVA in a prophylactic model (Melendez et al., 2007; Rzepecka et al., 2013). This model (Supplementary Fig. S1A) gives significant cell infiltration and a strong inflammatory response in the lungs during antigen challenge (Patel et al., 2005), and involves targeting of both the sensitization and challenge phases by the SMAs. Although we have previously reported that ES-62 inhibits mast cell degranulation in the lungs in this model (Melendez et al., 2007), the number of mast cells detectable in the bronchoalveolar lavage (BAL) is relatively very small and thus we focused on an alternative approach to assessing an anti-allergy effect, specifically on measuring the levels of more abundant inflammatory cell types that infiltrate the lungs during OVA challenge. Thus, as can be seen in Fig 3 the two SMAs successfully reduced eosinophil infiltration both in terms of the proportion (Fig. 3A) and absolute numbers (Fig. 3B) of cells as measured by BAL. Effects on neutrophils were less obvious, e.g., inhibition of neutrophil numbers (Fig. 3C) only reached statistical significance with SMA 12b employed at a 1 μg dose. No significant effect could be found on either macrophage or lymphocyte infiltration via BAL (results not shown). The protective effects of 11a and 12b were confirmed by histological analysis of H & E staining (Fig. 3D and E) and were corroborated by findings that, similar to ES-62 (Rzepecka et al., 2013), both SMAs reduced the mRNA levels of IL-4 (Fig. 3F) and IL-17 (Fig. 3G) which act to drive pathogenic Th2 responses in the lungs (Iwakura et al., 2011; Dias and Banerjee, 2012). The suppression of IL-17 is perhaps surprising in the context of the observed relatively weak inhibitory effect of the two SMAs on neutrophil influx of the lungs, given the role played by this cytokine in neutrophil recruitment (Ye et al., 2001), but the data may simply indicate that the levels of neutrophils in the lungs are not solely dependent on local levels of IL-17. At the same time, its targeting may reflect the important role of IL-17 in driving eosinophilic inflammation, including in asthma (Iwakura et al., 2011; Dias and Banerjee, 2012).

Finally, we also employed a therapeutic model (Supplementary Fig. S1B) of airway hyper-reactivity (Patel et al., 2005), which is more relevant to the clinical situation, e.g., in treating asthma exacerbations. In this OVA-based model, SMAs 11a and 12b showed evidence of inhibiting both the proportion (Fig. 3H) and absolute numbers (Fig. 3I) of neutrophils, but not eosinophils (results not shown) in the BAL. We also measured the levels of IL-4 and IL-17 mRNA in the lungs in this therapeutic model but although there was a tendency towards a reduction with respect to IL-4, no statistically significant changes were observed with either cytokine when mice were exposed to the two SMAs (results not shown). Again, the result obtained with IL-17 is more indicative of a correlation between local levels of this cytokine and eosinophil, rather than neutrophil, infiltration whilst the lack of a statistically significant reduction with regards to IL-4 in this therapeutic model may possibly reflect the Th2 polarisation existing prior to administration of the SMAs. Nevertheless, a statistically significant reduction in mRNA for IL-13 (Fig. 3J), another Th2 cytokine known to play a key role in asthma pathogenesis (Wills-Karp, 2004), was observed when a 10 μg dose of SMA 11a or 12b was employed. Furthermore, this inversely correlated with an increase in mRNA for the Th1-associated cytokine IFNγ (Fig. 3K) therefore indicating that the two SMAs were showing some evidence of mimicking ES-62 (Rzepecka et al., 2013) in reversing the polarity of the Th cell response.

Several parasitic nematode products, in addition to ES-62, including filarial nematode cystatin (Schnoeller et al., 2008) and a MIF homologue from *Anisakis simplex* (Park et al., 2009), have been reported to be active in protecting against OVA-induced airway hypersensitivity in mouse models. However as alluded to earlier, one potential problem in exploiting such immunomodulators for use in the clinic is their potential immunogenicity. In the case of ES-62, the active anti-inflammatory moiety is a nematode-specific post-translational modification (Harnett et al., 2008; Al-Riyami et al., 2013) and therefore this provided an opportunity to explore a small molecule mimetic approach. By taking this opportunity, we have now provided proof-of-principle that drug-like SMAs of a helminth product with anti-allergy properties can be designed. Furthermore, we have shown not only that these compounds mimic the parent molecule in offering protection in a prophylactic model of airway hyperresponsiveness, but that they can also be successfully employed in a therapeutic model. This strengthens the idea that ES-62-based SMAs represent a starting point in novel drug development for the treatment of asthma. The inhibitory effect on neutrophils observed in the therapeutic model is particularly interesting given the role played by these cells in chronic, steroid-resistant asthma, which is often very difficult to treat and where there is a clear unmet clinical need.

## Figures and Tables

**Supplementary Fig. S1:**
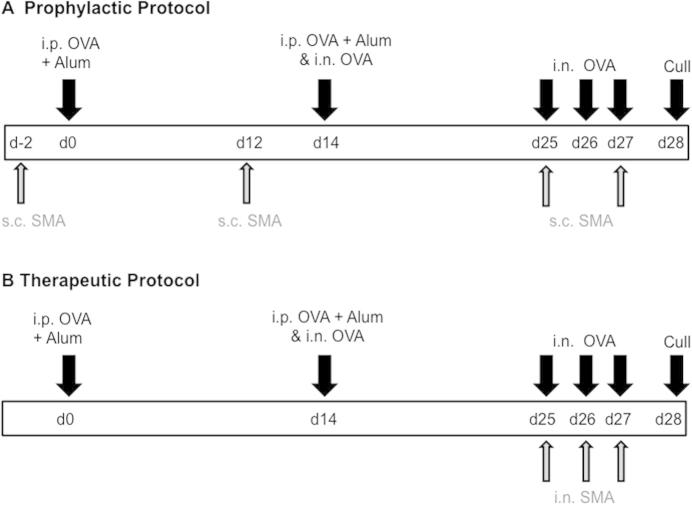
Model of ovalbumin (OVA)-induced airway hypersensitivity. Six to eight week old female BALB/c mice were sensitised by i.p. injection of 100 μg of OVA in 200 μl of 1% alum (Alhydrogel; Brenntag Biosector, Fredriksund, Denmark) on days (d) 0 and 14. Subsequently on day 14 mice were challenged by the intranasal (i.n.) route with 50 μg of OVA in 30 μl of PBS (endotoxin-free, Lonza, Slough, UK) after anaesthesia was induced with isoflurane. On days 25, 26 and 27 mice were anaesthetised and re-challenged i.n. with 50 μg of OVA in 30 μl of PBS. Control mice received PBS in place of OVA. (A) For the prophylactic model, 1 or 10 μg of SMAs 11a or 12b in 100 μl of PBS was administered by s.c. injection in the scruff of the neck on days −2, 12, 25 and 27. (B) For the therapeutic model, 1 or 10 μg of SMAs 11a or 12b in 30 μl of PBS was administered by the i.n. route, 1 h prior to OVA administration on days 25, 26 and 27. In both models, mice were subjected to euthanasia on day 28 by lethal i.p. injection of avertin (1,1,1-tribromoethanol) dissolved in iso-amyl alcohol and diluted 1/40, and bronchoalveolar lavage (BAL) and analyses undertaken as described in the text.

**Fig. 1:**
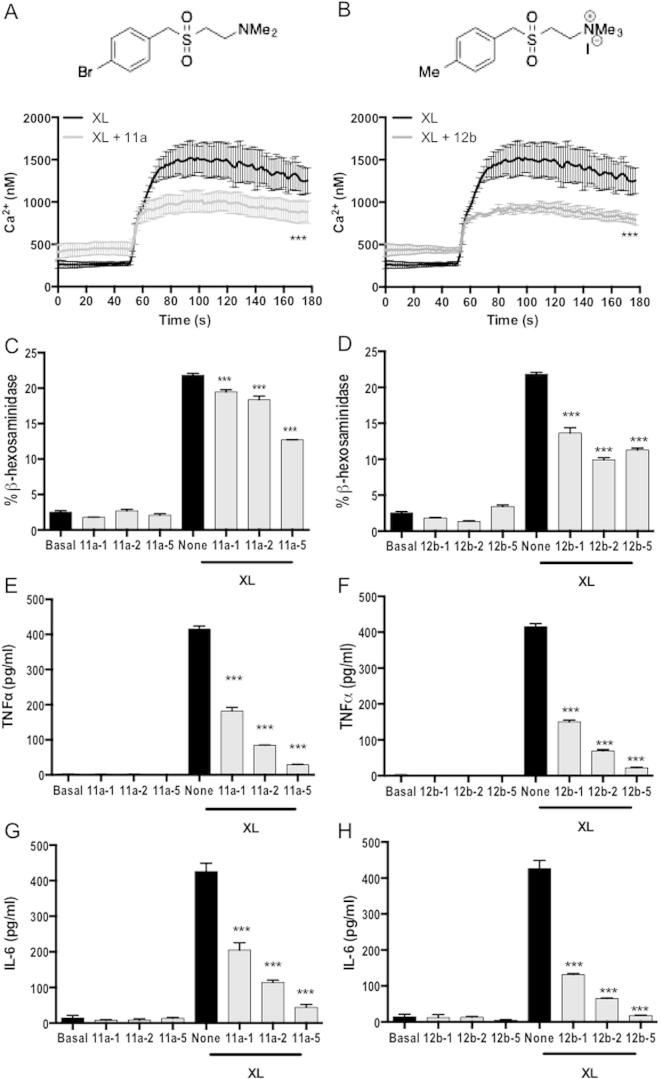
Small molecule analogues (SMAs) 11a and 12b of the immunomodulatory helminth product ES-62 inhibit mast cell calcium mobilisation, degranulation and cytokine production. (A, B) Structures of 11a and 12b and their effects on high affinity IgE receptor (FcεRI)-mediated calcium mobilisation. Measurement of calcium mobilisation was undertaken as previously described (Ball et al., 2013). Mouse bone marrow-derived mast cells were sensitised with murine anti-DNP IgE (0.5 μg/ml) in the presence and absence of SMA 11a or 12b (5 μg/ml) overnight at 37 °C. Following loading with Fura-2-acetyoxymethyl ester (Fura-2AM), the cells were stimulated at the 50 s time-point with DNP (0.5 μg/ml) to induce cross-linking (XL) of FcεR1 and intracellular calcium mobilisation and influx was then recorded in real time using excitation-emission ratios of 340/380 nm. Data are presented as the mean calcium values of triplicate samples and ^∗∗∗^*P* < 0.001 by ANOVA and Newman–Keuls multiple comparison test. (C, D) Peritoneal-derived mast cells and (E–H) Bone marrow-derived mast cells were pre-treated with the indicated concentrations of 11a and 12b (1, 2 or 5 μg/ml) during the IgE sensitisation phase and then degranulation was measured by release of β-hexosaminidase as described previously (Melendez et al., 2007; Ball et al., 2013) 1 h following crosslinking of the IgE-bound FcεRI (XL) or not (C, D). (E, F) TNFα and (G, H) IL-6 release were measured by ELISA as described previously (Melendez et al., 2007; Ball et al., 2013) 24 h following crosslinking. Data are presented as means ± S.D. where *n *= 3 and analysis is by one-way ANOVA and Newman–Keuls post test where ^∗∗∗^*P *< 0.001. All data are representative of at least three independent experiments.

**Fig. 2:**
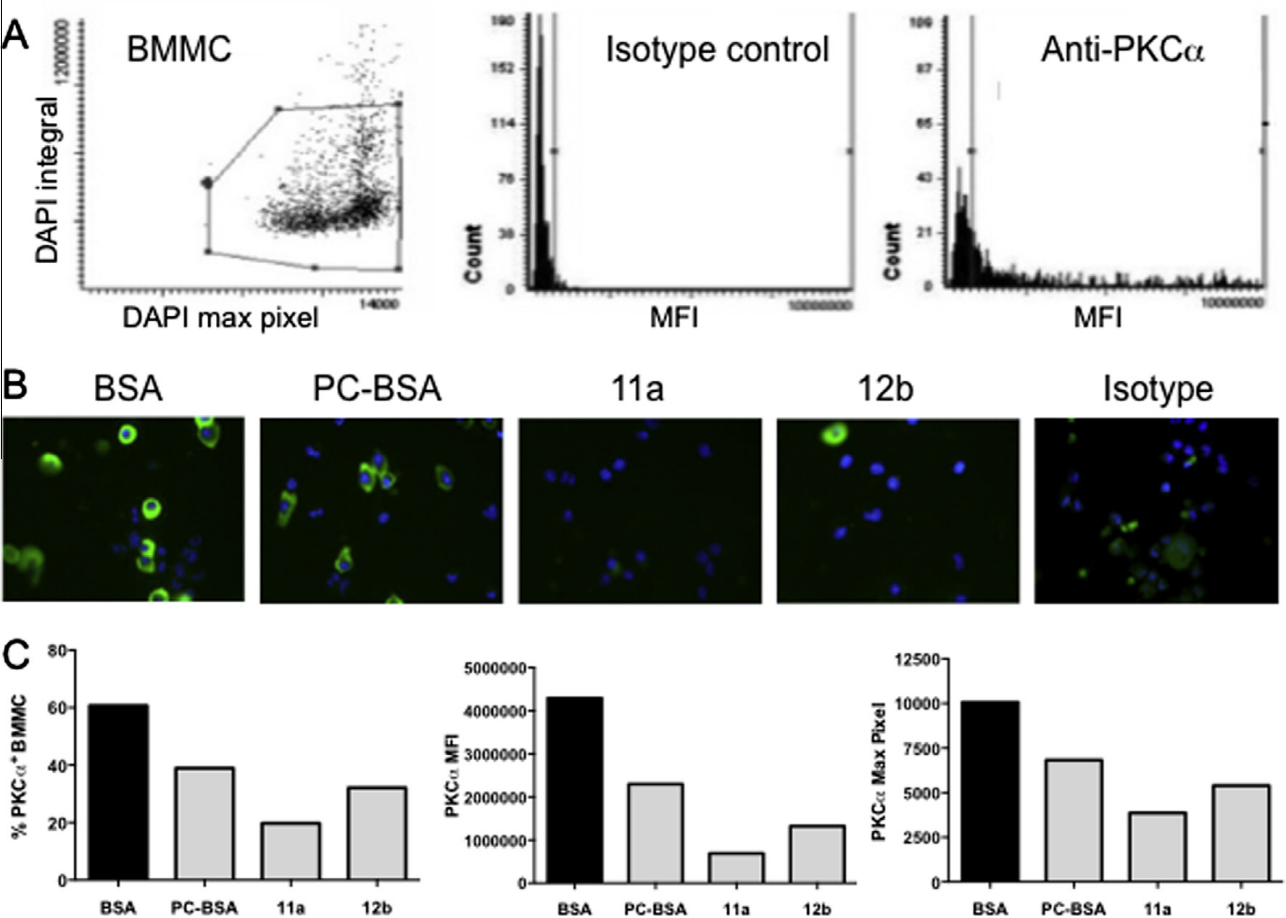
Small molecule analogues (SMAs) 11a and 12b of the immunomodulatory helminth product ES-62 induce Protein Kinase C alpha (PKCα) down-regulation in mouse bone marrow-derived mast cells (BMMCs). PKCα expression was measured by laser scanning cytometry (LSC) as described previously (McGrath et al., 2011). BMMCs were sensitised with anti-DNP-IgE overnight in the presence of 1 μg/ml of BSA, phosphorylcholine (PC)-BSA, SMA 11a or SMA 12b prior to fixing, permeabilising and staining of PKCα expression using anti-mouse PKCα antibody. (A) Exemplar plots of the gating strategy for quantitative analysis of BMMCs (gated on DAPI staining of their nuclei) for PKCα expression mean fluorescence integral (MFI) relative to an IgG2a isotype control. (B) The relocation feature of the LSC generates representative images (40×) of PKCα (Alexa 488; green) expression by BMMCs from each treatment group, counterstained with DAPI (blue) to identify the cell nucleus. Images were captured using a Hamamatsu Orca ER digital camera and the OpenLab 3.0.9 digital imaging program (Improvision). (C) Analysis by Wincyte software of at least 2000 BMMCs under each treatment condition allowed quantitation of the percentage of PKCα^+^ BMMCs in each group, the MFI value of PKCα expression by BMMCs and the maximum (max) pixel value of PKCα expression by BMMCs.

**Fig. 3:**
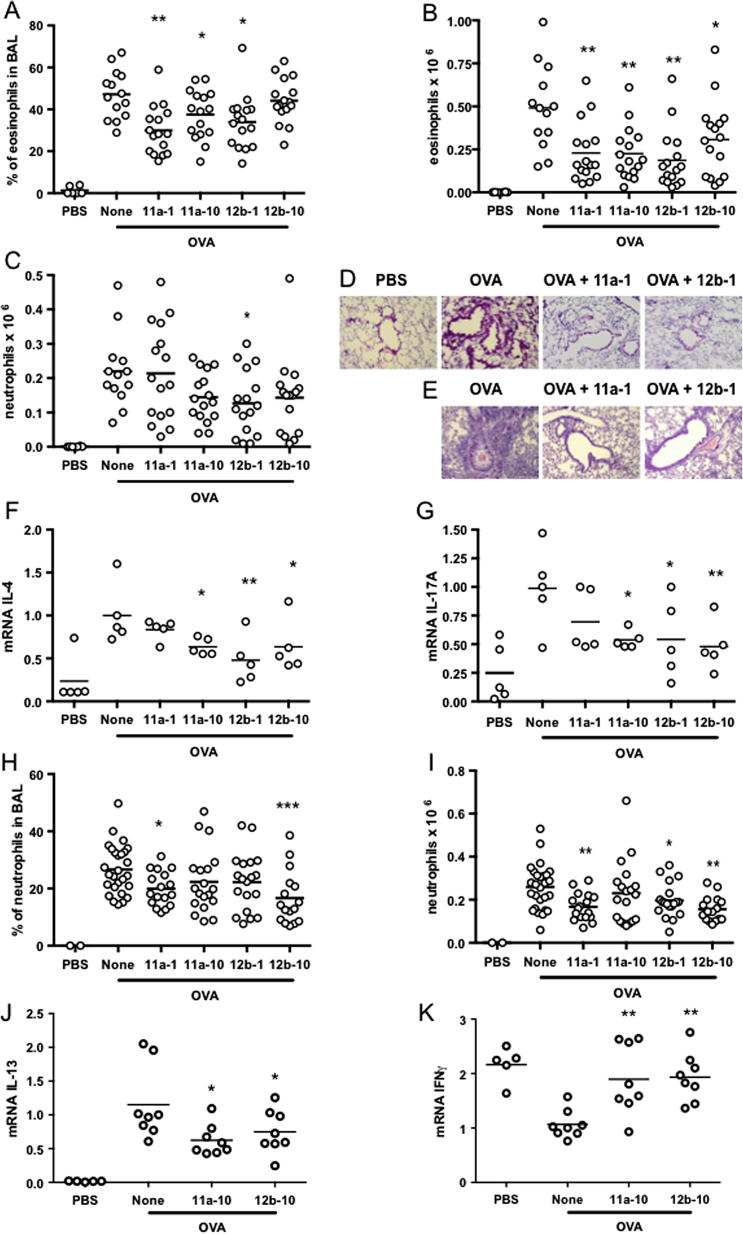
Small molecule analogues (SMAs) 11a and 12b of the immunomodulatory helminth product ES-62 suppress ovalbumin (OVA)-induced airway hyper-responsiveness (OAH). BALB/c mice undergoing OAH (OVA) were treated with SMAs 11a (1 or 10 μg/dose) or 12b (1 or 10 μg/dose) either prophylactically (s.c.; A–G) as described in Supplementary Fig. S1A and previously (Rzepecka et al., 2013) or therapeutically (intra-nasal; H–K) where mice only received SMAs 1 h before OVA challenge on days 25, 26 and 27 of the experiment as described in Supplementary Fig. S1B. Mice were assessed at sacrifice via bronchoalveolar lavage for lung eosinophil (A, B) or neutrophil infiltration (C, H, I) and lung pathology as indicated by H & E staining from two independent models (D and E; PBS represents control mice). In A–C, data are pooled from three independent experiments and for H and I, data are pooled from four independent experiments. Analysis of IL-4 (F), IL-17 (G) IL-13 (J) and IFNγ (K) mRNA levels in the lungs from the various treatment groups from a single prophylactic (F, G) or therapeutic (J, K) experiment are shown. Real time quantitative Reverse Transcription-PCR procedures were carried out as described previously (Rzepecka et al., 2013) and according to the manufacturer’s instructions (Applied Biosystems, Carlsbad CA, USA) using assay kits, Mm 00445259_m1 (IL-4), Mm00439619_m1 (IL-17), Mm00434204_m1 (IL-13), Mm 01168134_ml (IFNγ) and Mm 99999915_g1 (GAPDH). All samples were examined in triplicate and data analysed by StepOne software using the comparative C_T_ (ΔΔC_T_) (Applied Biosystems) with values for samples being normalised to the reference reporter GAPDH. All data represent responses from individual mice in the indicated groups. Where relevant, analysis was by one-way ANOVA using Bonferroni’s post test; ^∗^*P* < 0.05, ^∗∗^*P* < 0.01 and ^∗∗∗^*P* < 0.001, respectively. All procedures were conducted in accordance with home office UK animal guidelines and with the approval of the local ethical committees.
